# Assessment of awareness, usage, and attitudes toward nicotine pouches in Saudi Arabia: a cross-sectional study

**DOI:** 10.3389/fpubh.2026.1896313

**Published:** 2026-08-27

**Authors:** Hussain Khalifa Aljumah, Hassan Ali Alradhi, Mujtaba Abbas Alzuwayr, Manal Yousef Alturaifi, Maryam Ali Alamer, Mohammed Naji Albannay, Abdullah Almaqhawi

**Affiliations:** 1 College of Medicine, King Faisal University, Al-Ahsa, Saudi Arabia; 2 Faculty of Medicine, Jazan University, Jazan, Saudi Arabia; 3 College of Applied Medical Sciences, King Saud bin Abdulaziz University for Health Sciences, Al-Ahsa, Saudi Arabia; 4 Department of Family and Community Medicine, College of Medicine, King Faisal University, Al-Ahsa, Saudi Arabia

**Keywords:** attitudes, awareness, nicotine pouches, Saudi Arabia, smokeless tobacco, use

## Abstract

**Background:**

Nicotine pouches (NPs) are emerging smokeless nicotine products marketed as tobacco-free alternatives to traditional smokeless tobacco products. Despite their growing popularity, particularly among youth, limited research exists on their awareness, usage, and attitudes in Saudi Arabia.

**Objectives:**

This study aimed to assess awareness, usage patterns, and attitudes toward NPs among adults in Saudi Arabia.

**Methods:**

A cross-sectional, online survey targeting Arabic-speaking adults was conducted between 6 March and 6 June 2025. The self-administered questionnaire covered demographics, NP awareness, usage, and attitudes. Descriptive statistics and logistic regression were used to analyze predictors of awareness, usage, and favorable attitudes.

**Results:**

Among 1,004 participants, 69.9% had heard of nicotine pouches, yet comprehensive knowledge regarding their composition and regulatory aspects remains limited. NP use was reported by 25.5%, with the primary motivation for continued use being for smoking cessation (62.4%). Higher awareness was significantly associated with younger age (aOR = 0.452, 95% CI: 0.247–0.830, *p* = 0.010), male gender (aOR = 7.610, 95% CI: 2.462–23.529, *p* < 0.001), and residence in the Western, Central, or Southern regions (all *p* < 0.05). Use was also more likely among younger males, employed individuals, and those with higher income. Regarding overall attitudes, 62.9% of participants perceived NPs as easier to use and more discreet than traditional tobacco products, 49.9% believed they could help people quit smoking, and 49.3% considered them safer than e-cigarettes. Conversely, 69.6% believed NPs are addictive, 68.9% expressed concern about oral health risks, and 62.1% agreed that their long-term health effects are not well understood. Favorable attitudes were more common among males, ex-smokers, and current NP users (*p* < 0.001).

**Conclusion:**

Awareness and use of NPs are rising in Saudi Arabia, especially among young males and ex-smokers. Despite their potential role in harm reduction, misconceptions and unclear perceptions persist. These findings highlight the need for targeted public education and appropriate regulatory oversight to manage the public health impact of NP use.

## 1 Introduction

Smokeless tobacco (SLT) includes a range of products consumed without combustion, such as chewing tobacco, snuff, and snus. They are typically used orally or nasally and are often perceived as less harmful than smoked tobacco as they do not release the toxic chemicals during tobacco combustion (1). SLT has been linked to a range of adverse health outcomes, especially oral and dental diseases, that disproportionately affect lower-income populations (2). Globally, SLT remains a major public health threat, accounting for an estimated 350,000 deaths annually worldwide (36).

In recent years, oral nicotine pouches (NPs) have emerged as a novel category of smokeless nicotine products. Unlike traditional SLT forms, NPs are tobacco-free and consist of a cellulose-based pouch filled with either naturally derived or synthetic nicotine. While they deliver nicotine in amounts comparable to SLT, they contain substantially lower concentrations of harmful compounds (3, 8, 30). NPs were introduced to the U.S. market in 2016, with sales rising from just over 163,178 units in 2016 to nearly 46 million by 2020 (3, 4). Their popularity has been fueled by diverse flavors, varying nicotine strengths, and aggressive online marketing with products available in a wide range of appealing options such as black cherry, coffee, peppermint, and citrus (3, 4). NPs are often promoted as odorless, discreet, and socially acceptable alternatives to cigarettes or vaping products, features that particularly appeal to younger demographics. Moreover, they are advertised across a range of platforms, including social media, and are frequently described as “tobacco-free” and containing “synthetic nicotine,” while being portrayed as more practical than traditional cigarettes or e-cigarettes (4–7).

While NPs may appear to be a safer alternative, given toxicological evidence indicating they are less harmful than snus, combustible tobacco, and some e-cigarettes, their long-term health effects remain uncertain, particularly when compared to other smokeless nicotine products (8–10). Usage rates of NPs among young adults have differed across studies. According to the 2021 National Youth Tobacco Survey, 3.0% of high school students reported having tried NPs (11). Regionally, a cross-sectional survey in Riyadh found that 14.2% had tried them, highlighting their increasing visibility and use in Saudi Arabia (12). Various types of NPs, including globally recognized brands like “Velo” and locally produced options such as “DZRT,” are also available and used in Saudi Arabia (13).

Although NPs are becoming widely available, research on their awareness and use in Saudi Arabia remains limited. Therefore, this study aims to evaluate the awareness, use behaviors, and attitudes regarding NPs among adults in Saudi Arabia.

## 2 Methodology

This cross-sectional study was carried out over 3 months (from 6 March to 6 June 2025) using a self-administered online questionnaire.

### 2.1 Inclusion and exclusion criteria

The target population was Arabic-speaking adults aged 18 years or older residing in Saudi Arabia. Individuals younger than 18 years of age or unable to understand Arabic were excluded. Participants who provided incomplete responses to specific sub-questions were not excluded. Missing data were minimal (<1% of the total sample) and strictly confined to secondary descriptive questions directed at current users; all 1,004 participants provided complete data for the primary variables included in the regression analyses.

### 2.2 Sampling and sample size calculation

The sample size was determined using Cochran’s formula $n=(\frac{z^{2}\mathrm{pq}}{d^{2}})$ , with an estimated proportion set at 50, 95% level of confidence, 5% degree of accuracy, and an estimated population of about 28 million adults, a minimum sample size of 385 was calculated. To enhance the reliability and accuracy of the findings, additional participants were recruited for the study. A non-random convenience sampling strategy was employed, leveraging social media platforms to reach eligible adult residents across Saudi Arabia.

### 2.3 Data collection tool

The questionnaire was adapted from previously published studies on novel nicotine products (Supplementary file 1) (14–17). Items were curated from prior instruments and explicitly modified to align with the objectives of this study and the cultural context of Saudi Arabia. Participants were provided with a study description and an image of NPs. The final questionnaire comprised 36 items across four sections: (1) Demographics and smoking status; (2) Awareness and knowledge (composite score out of 5); (3) Usage behaviors and future intentions among current NP users; and (4) Attitudes toward NPs. The attitudes section included 13 statements rated on a 5-point Likert scale, with unfavorable public-health statements reverse-scored so that higher scores reflected a more favorable perception of NP use. The highest age category in the questionnaire was set at ‘Over 45 years.’ This was a deliberate design choice to ensure sufficient statistical power for the regression analyses, given the anticipated lower representation of older demographics in the study population.

### 2.4 Validity and reliability of the questionnaire

The questionnaire underwent expert review by two family medicine faculty members to ensure content accuracy and clarity. Initially, it was developed in English and was translated into Arabic by two bilingual researchers. To confirm the accuracy of the translation, a back-translation to English was performed. A pilot study was conducted with 20 participants to assess clarity and comprehension; these pilot responses were excluded from the final analysis. To assess internal consistency, Cronbach’s alpha was calculated for each domain, resulting in values of 0.76 for awareness and 0.83 for attitudes, reflecting good reliability across the questionnaire domains.

### 2.5 Data collection procedure

The questionnaire was created in Google Forms and distributed via multiple social media platforms, including WhatsApp, X (formerly Twitter), Instagram, and Telegram.

To ensure data protection and confidentiality, all responses were anonymized. The data were stored in a password-protected Google Sheet accessible only to the research team, and no personally identifiable information, such as names or IP addresses, was collected.

### 2.6 Ethical considerations

Ethical approval was obtained from the Research Ethics Committee at King Faisal University (reference number: KFU-REC-2025-MAR-ETHICS3222). Participation was voluntary, and informed consent was obtained electronically prior to initiating the survey. The study adhered to Strengthening the Reporting of Observational Studies in Epidemiology (STROBE) guidelines for cross-sectional studies (Supplementary file 2) (18).

### 2.7 Statistical analysis

Data were analyzed using IBM SPSS Statistics version 29 and R. Categorical variables were summarized using frequencies and percentages, while continuous variables were summarized using means and standard deviations. Normality was assessed using histograms, Q–Q plots, and the Shapiro–Wilk test. Given the large sample size (*n* = 1,004), independent t-tests and one-way ANOVA were considered appropriate under the Central Limit Theorem. Independent t-tests and one-way ANOVA were used to compare mean attitude scores across subgroups. Logistic regression analyses were conducted to identify predictors of higher awareness and current NP use. Variables included in the models were age, gender, nationality, education level, employment status, marital status, income, region, chronic disease status, and smoking history. Adjusted odds ratios (aOR) with 95% confidence intervals (CI) were reported. The adequacy of the multivariable logistic regression models was ensured through *a priori* selection of conceptually distinct sociodemographic covariates and a large sample size that comfortably exceeded standard events-per-variable thresholds, thereby preventing model overfitting. A *p*-value < 0.05 was considered statistically significant.

## 3 Results

### 3.1 Participant characteristics

A total of 1,004 participants were included in the final analysis. The sociodemographic characteristics of the participants are detailed in Table 1. More than half of the respondents were in the 18–25 years age group (53.1%) and were males (59.6%). The vast majority were Saudi nationals (95.7%), held a bachelor’s degree (67.3%), and were either students (43.6%) or employed (40.9%). Regarding marital and economic status, over half of the respondents were single (62.0%) and reported a monthly income of less than 5,000 SAR (51.8%).

**Table 1:** Sociodemographic characteristics of participants (*n* = 1,004).

Variable	Category	Frequency *n* (%)
Age group	18–25 Years	533 (53.1%)
	26–35 Years	211 (21.0%)
	36–45 Years	119 (11.9%)
	>45 Years	141 (14.0%)
Gender	Female	406 (40.4%)
	Male	598 (59.6%)
Nationality	Non-Saudi	43 (4.3%)
	Saudi	961 (95.7%)
Educational level	Up to secondary education	246 (24.5%)
	Intermediate education	14 (1.4%)
	Bachelor’s	676 (67.3%)
	Master’s/PhD	68 (6.8%)
Employment status	Not employed	99 (9.9%)
	Student	438 (43.6%)
	Employee	411 (40.9%)
	Retired	56 (5.6%)
Marital status	Single	622 (62.0%)
	Married	367 (36.6%)
	Divorced/widow	15 (1.5%)
Monthly income	<5,000 SAR	520 (51.8%)
	5,000–10,000 SAR	185 (18.4%)
	10,000–15,000 SAR	141 (14.0%)
	>15,000 SAR	158 (15.7%)
Presence of chronic disease	No	852 (84.9%)
	Yes	152 (15.1%)
Type of chronic disease*	Chronic Hypertension	52 (34.2%)
	Diabetes	50 (32.9%)
	Asthma	15 (9.9%)
	COPD	12 (7.9%)
	Allergic Rhinitis/Eczema	4 (2.6%)
	Other	19 (12.5%)

Data are presented as frequencies and percentages, *n* (%). *Percentages for specific chronic diseases are calculated based on the sub-sample of participants reporting a comorbidity (*n* = 152).

**Figure 1:**
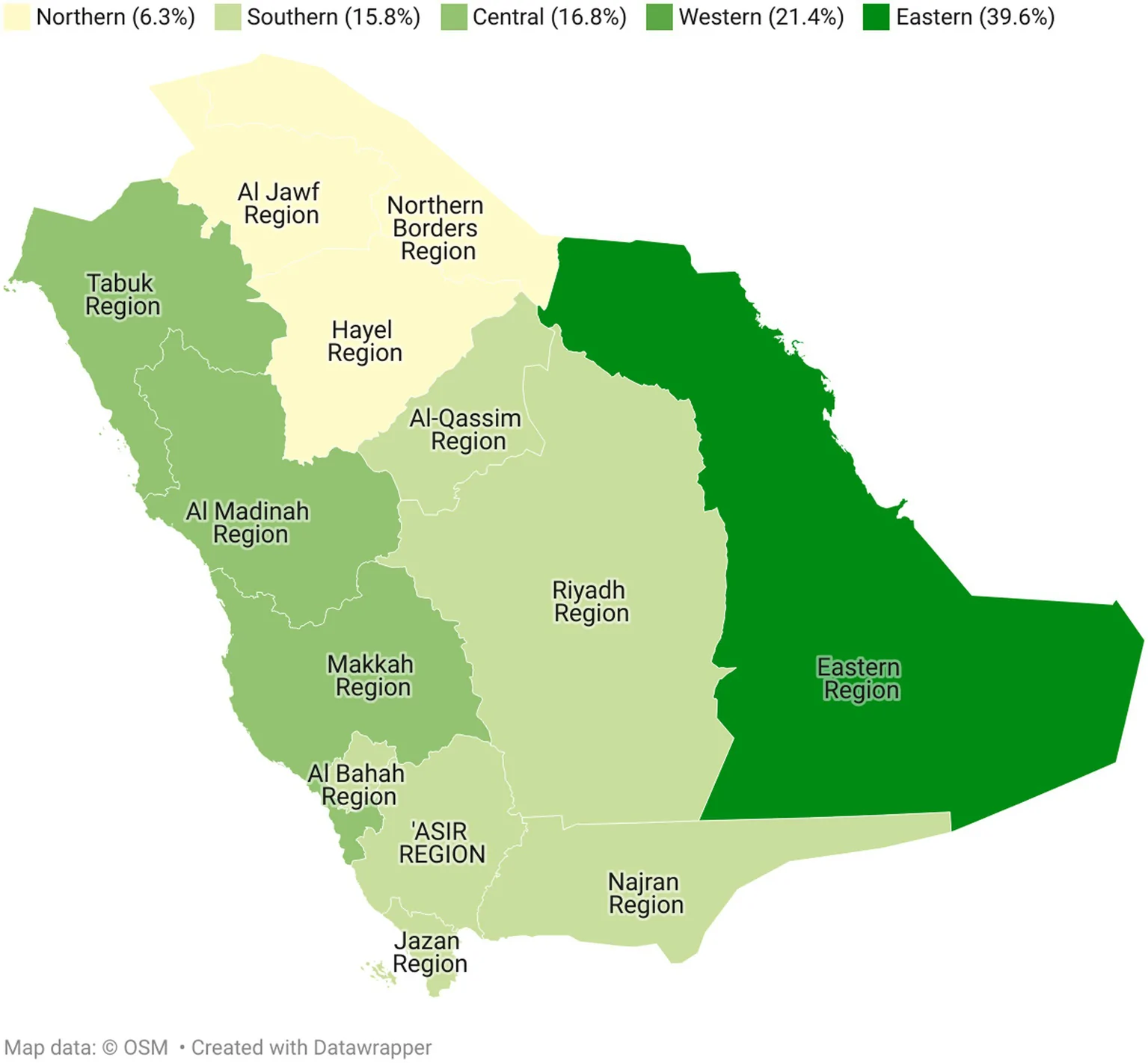
Geographical distribution of participants (*n* = 1,004). Source: OpenStreetMap contributors (via Datawrapper). Map data is available from https://www.openstreetmap.org. License: Open Data Commons Open Database License (ODbL).

Participants were geographically distributed across all regions, with the largest proportions being from the Eastern (39.6%) and the Western (21.4%) regions, followed by other regions, as shown in Figure 1. A history of chronic diseases was reported by 15.1% of the participants, with chronic hypertension (34.2%) and diabetes (32.9%) being the most common.

**Figure 2:**
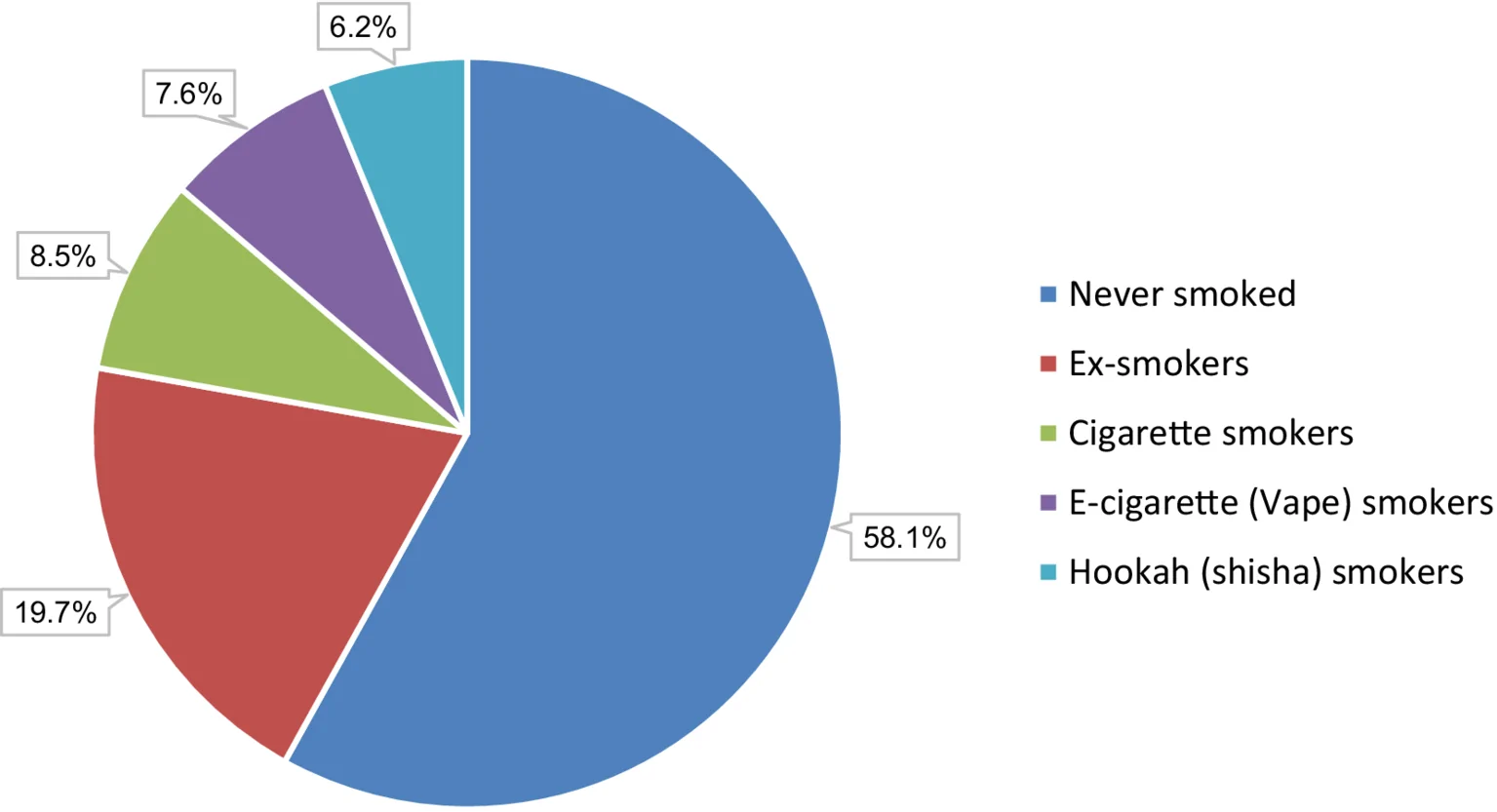
Smoking status among participants (*n* = 1,004).

Regarding smoking status, most participants (58.1%) had never smoked, while 19.7% were ex-smokers (Figure 2). Among current users, 8.5% smoked cigarettes, 7.6% used e-cigarettes, and 6.2% smoked hookah/shisha. Among current or former smokers (*n* = 421), 29.5% reported smoking for less than 5 years, 30.2% for 5 to 10 years, and 18.3% for more than 15 years.

### 3.2 Awareness and usage patterns

**Figure 3:**
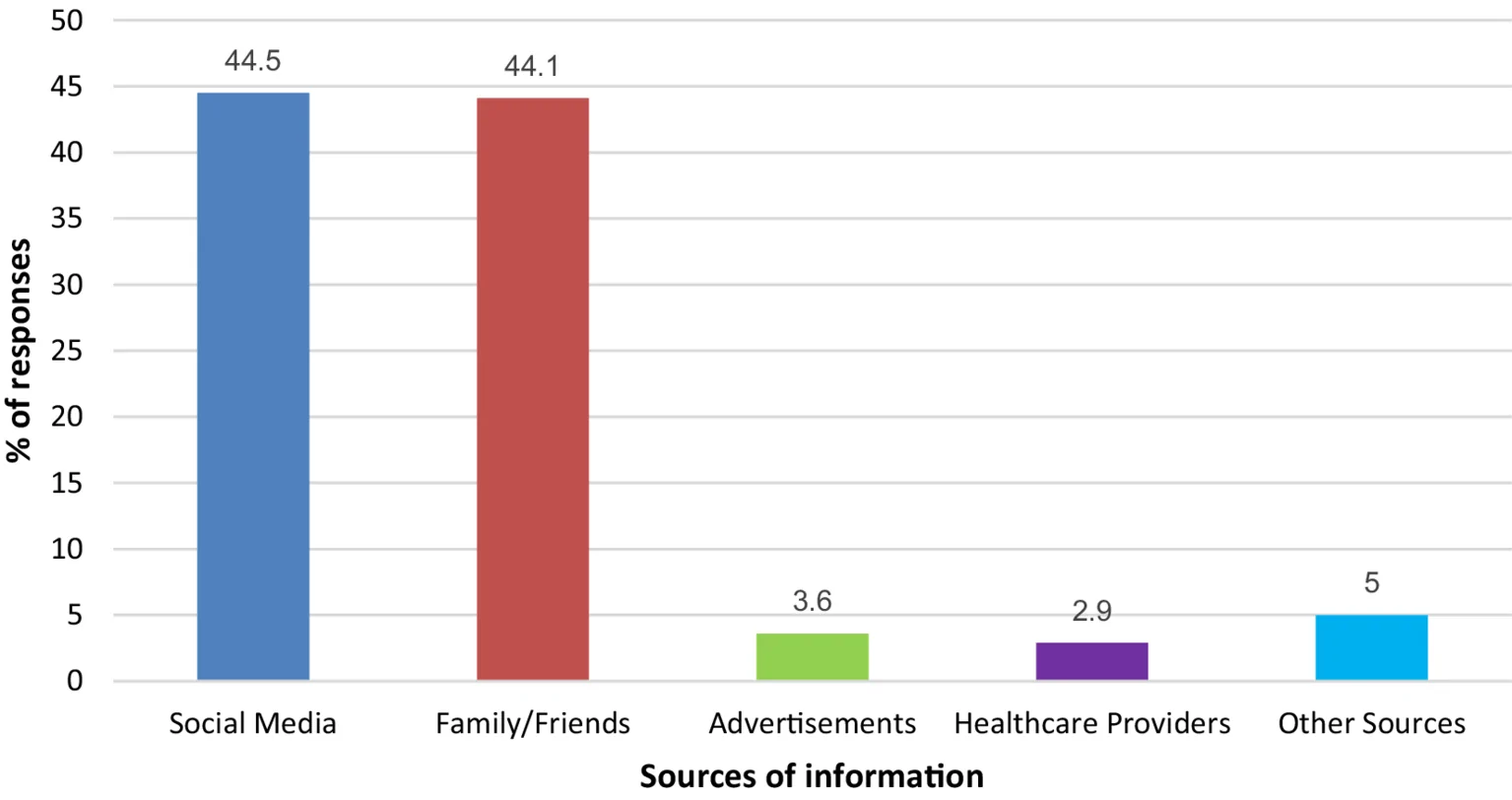
Sources of information regarding nicotine pouches (*n* = 702).

Assessment of participant awareness revealed that 69.9% of respondents had heard of NPs, and 69.5% were familiar with smokeless tobacco products. Regarding specific product characteristics, 64.0% knew that NPs are available in various flavors and strengths. However, only 36.9% were aware that they are tobacco-free, and 48.0% knew about the legal age restrictions (Table 2). The most common sources of information about NPs are summarized in Figure 3. Overall baseline knowledge was further quantified, yielding a mean awareness score of 2.9 ± 1.7 out of a maximum possible score of 5 (Table 2).

**Table 2:** Awareness and usage patterns of nicotine pouches (*n* = 1,004).

Item	Response	Frequency *n* (%)
Awareness of nicotine pouches		
Awareness of smokeless tobacco products	No	306 (30.5%)
	Yes	698 (69.5%)
Awareness of nicotine pouches	No	302 (30.1%)
	Yes	702 (69.9%)
Knowledge of available flavors and strengths	No	361 (36.0%)
	Yes	643 (64.0%)
Knowledge of the tobacco-free nature	No	634 (63.1%)
	Yes	370 (36.9%)
Awareness of age restrictions on purchasing and consumption	No	522 (52.0%)
	Yes	482 (48.0%)
Mean awareness score	Mean (SD)	2.9 (1.7)
Usage of nicotine pouches		
Current nicotine pouch use	No	749 (74.5%)
	Yes	255 (25.5%)
Average daily consumption	<5 Pouches	84 (34.3%)
	5–10 Pouches	118 (48.2%)
	>10 Pouches	43 (17.6%)
Likelihood of continued use over next year	Very Unlikely	38 (15.3%)
	Unlikely	38 (15.3%)
	Neutral	30 (12.0%)
	Likely	81 (32.5%)
	Very likely	62 (24.9%)
Primary motivation for continued use	To quit smoking	159 (63.3%)
	Recreational/nicotine addiction	39 (15.5%)
	Relieve stress/depression	28 (11.2%)
	No specific reason	22 (8.8%)
	Taste	3 (1.2%)
Concurrent use of other nicotine products	No	153 (61.0%)
	Sometimes	63 (25.1%)
	Yes	35 (13.9%)
Self-reported cravings	No	74 (29.0%)
	Sometimes	103 (40.4%)
	Yes	78 (30.6%)

Data are presented as frequencies and percentages, *n* (%). Sub-sample totals for specific follow-up questions directed at current users may not sum to exactly 255 due to incomplete participant responses for those individual items. Percentages for these specific items represent valid percentages based on the actual number of respondents for each question.

Regarding NP usage, 25.5% (*n* = 255) of the participants currently use NPs. Longitudinal utilization indicators and product experiences among current users are detailed in Table 2. In terms of future intentions to use NPs, more than half of current users indicated an inclination to maintain consumption over the coming year, with 31.8% of users reporting they were likely and 24.3% reporting they were very likely to continue. The primary motivation for continued use was to quit smoking (62.4%). While 60.0% of users exclusively utilized NPs, concurrent dual-use with other nicotine products was noted, with 24.7% reporting occasional dual-use and 13.7% reporting regular dual-use. Additionally, self-reported cravings were common, with 30.6% of users reporting regular increased desire for more pouches, while 40.4% experienced occasional cravings.

### 3.3 Attitudes and associated factors

**Figure 4:**
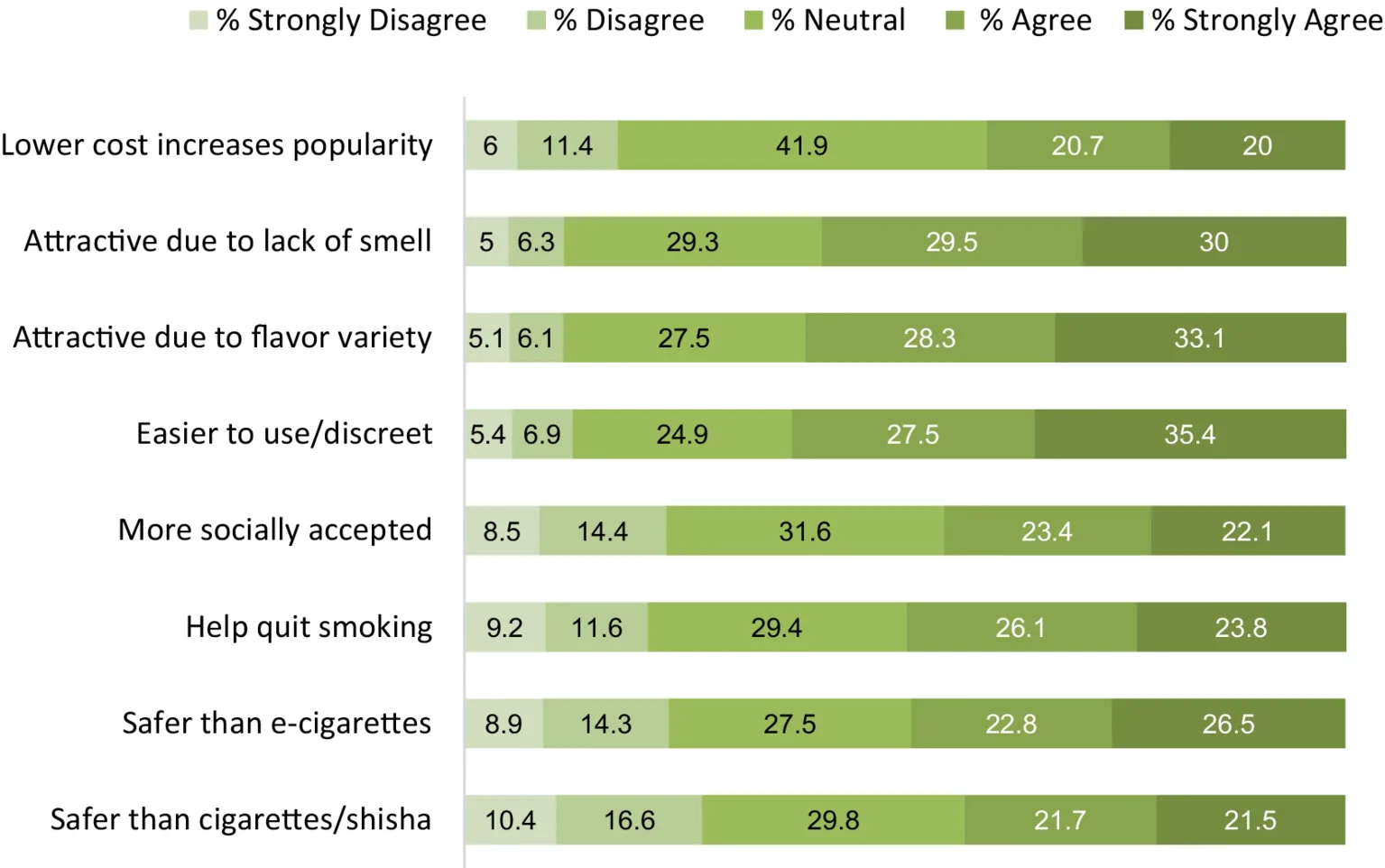
Favorable attitudes toward nicotine pouches (*n* = 1,004).

**Figure 5:**
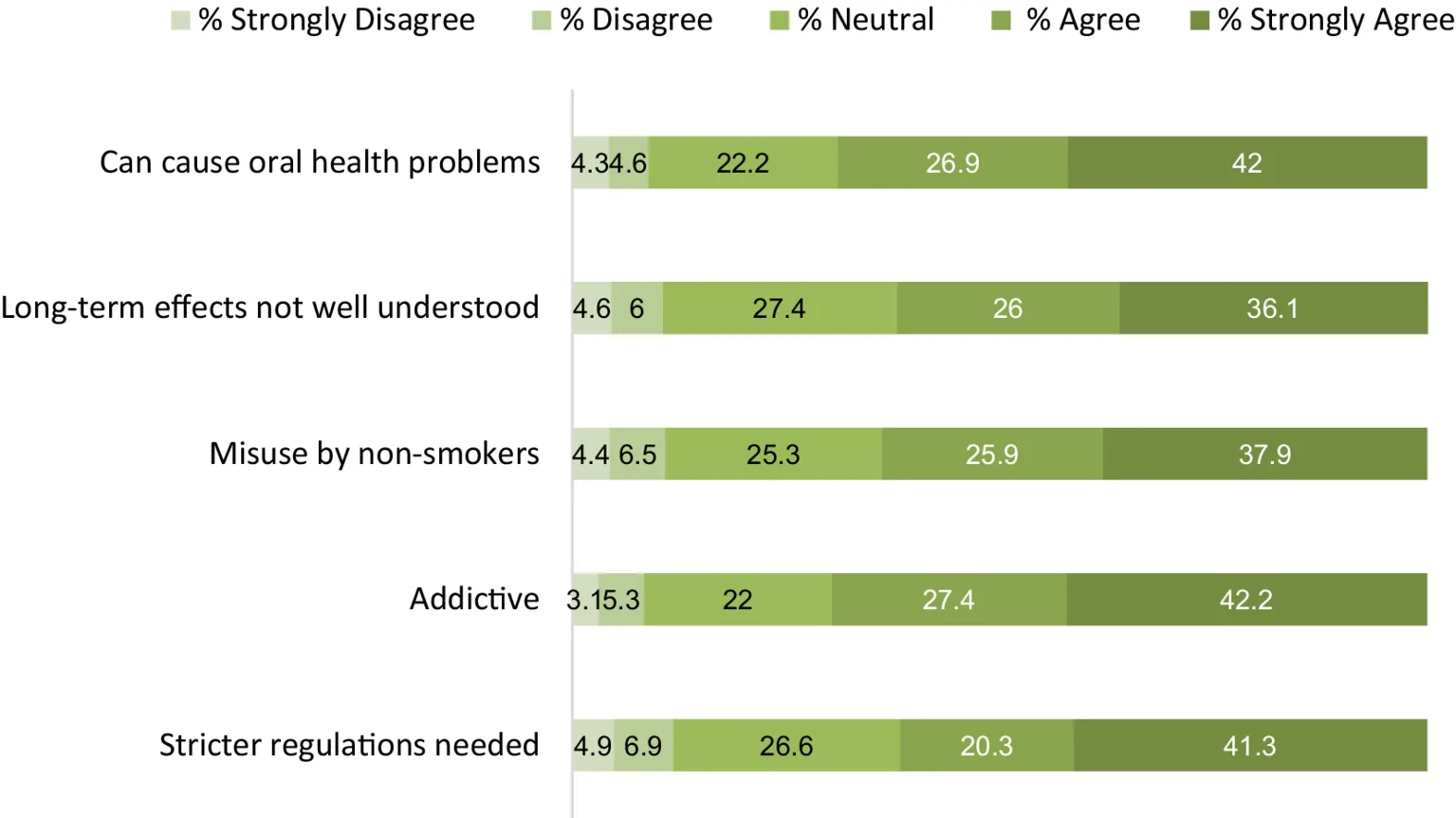
Unfavorable attitudes toward NPs (*n* = 1,004).

Participants’ attitudes toward NPs were characterized by a contrast of perceived practical benefits and significant health concerns (Figures 4, 5).

Regarding favorable attitudes, most respondents found NPs’ features appealing or beneficial (Figure 4). Most respondents appreciated the convenience and discretion compared to traditional tobacco products (62.9%), as well as the appeal of various flavors (61.4%). In addition, nearly half of the study population framed NPs as a potential harm-reduction tool, expressing the belief that pouches could facilitate smoking cessation (49.9%) or offer a safer alternative to e-cigarettes (49.3%).

Conversely, substantial unfavorable attitudes and concerns were reported regarding the risks of NPs (Figure 5). The primary concerns cited by participants were the addictive nature of the products (69.6%) and the potential for oral health complications, such as gum disease (68.9%). Furthermore, a strong majority recognized that the long-term health effects of these products are not well understood (62.1%) and supported the implementation of stricter regulations (61.6%).

Male participants showed significantly more positive attitudes than female participants (*p* < 0.001), and individuals in the 26–35 age group (*p* < 0.001) (Table 3). Employment status and geographic region also showed significant differences, with employed individuals and residents of the Northern region reporting the highest mean attitude scores.

**Table 3:** Bivariate analysis of factors associated with favorable attitudes toward nicotine pouches.

Variable	Category	Mean (SD)	*p*-value
Age group	18–25 years	40.01 (3.92)	<0.001^***b***^
	26–35 years	41.02 (3.51)	
	36–45 years	40.51 (4.19)	
	>45 years	38.90 (4.22)	
Gender	Female	38.99 (3.75)	<0.001^***a***^
	Male	40.90 (3.92)	
Nationality	Non-Saudi	40.00 (3.31)	0.832^a^
	Saudi	40.13 (3.99)	
Educational level	Up to secondary education	39.92 (4.14)	0.844^b^
	Intermediate education	40.43 (4.29)	
	Bachelor’s	40.17 (3.94)	
	Master’s/PhD	40.16 (3.54)	
Residential area	Eastern	39.72 (3.99)	0.005^***b***^
	Western	40.55 (3.75)	
	Central	40.59 (3.81)	
	Southern	39.67 (4.09)	
	Northern	41.11 (4.21)	
Employment status	Not employed	40.32 (3.65)	<0.001^***b***^
	Student	39.61 (3.93)	
	Employee	40.86 (3.88)	
	Retired	38.41 (4.33)	
Marital status	Single	40.17 (3.87)	0.888^b^
	Married	40.05 (4.12)	
	Divorced/widow	39.93 (4.13)	
Monthly income	<5,000 SAR	39.92 (3.86)	0.340^b^
	5,000–10,000 SAR	40.37 (3.69)	
	10,000–15,000 SAR	40.48 (4.08)	
	>15,000 SAR	40.20 (4.46)	
Presence of chronic disease	No	40.17 (3.89)	0.350^a^
	Yes	39.85 (4.34)	
Smoking status	Never smoked	39.06 (3.93)	<0.001^***b***^
	Ex-smoker	42.12 (3.65)	
	Smoke cigarette	41.69 (3.57)	
	Smoke E-cigarette (vaping)	40.88 (3.33)	
	Smoke hookah	40.73 (2.82)	
Smoking duration	<5 years	41.37 (3.44)	0.043^***b***^
	5–10 years	42.35 (3.67)	
	11–15 years	41.72 (3.53)	
	>15 years	41.05 (3.23)	
Awareness of smokeless tobacco products	No	39.33 (4.02)	<0.001^***a***^
	Yes	40.47 (3.89)	
Awareness of nicotine pouches	No	38.66 (3.81)	<0.001^***a***^
	Yes	40.76 (3.86)	
Current nicotine pouch use	No	39.26 (3.80)	<0.001^***b***^
	Yes	42.65 (3.27)	

Data are presented as mean (standard deviation). Higher attitude scores indicate a more favorable perception of nicotine pouches. (^a^) Independent *T* Test, (^b^) ANOVA.

Personal experience with tobacco and alternative nicotine products was a strong driver of favorable perceptions. Current nicotine pouch users, ex-smokers, and individuals with prior awareness of smokeless tobacco products had significantly higher positive attitude scores than non-users and never-smokers (all *p* < 0.001). Conversely, variables such as nationality, educational level, marital status, and monthly income did not significantly affect overall attitude scores.

### 3.4 Predictors of awareness and use

Multivariable logistic regression analyses were performed to identify independent sociodemographic and behavioral predictors of nicotine pouch awareness (Table 4) and current use (Table 5).

**Table 4:** Multivariable predictors of nicotine pouch awareness.

Variable	*B*	aOR	95% CI for aOR	*p*-value
Age	−0.793	0.452	0.247–0.830	0.010
Gender (male)	2.030	7.610	2.462–23.529	<0.001
Nationality (Saudi)	1.107	3.027	0.318–28.819	0.335
Higher educational level	0.209	1.233	0.812–1.872	0.325
Region (Eastern)	Ref	1	Ref	0.036
Region (Western)	1.441	4.226	1.325–13.479	0.015
Region (Central)	1.295	3.653	1.066–12.520	0.039
Region (Southern)	2.194	8.971	1.013–79.449	0.049
Region (Northern)	0.828	2.289	0.448–11.708	0.320
Employment status (employee)	0.149	1.161	0.592–2.277	0.664
Marital status (married)	−0.201	0.818	0.314–2.130	0.681
Higher monthly income	−0.106	0.899	0.555–1.458	0.667
Chronic disease (yes)	−0.820	0.440	0.170–1.144	0.092
Currently smoker (yes)	0.061	1.062	0.705–1.602	0.773
Smoking duration (up to 15 Yrs)	−0.529	0.589	0.386–0.900	0.014

aOR, Adjusted Odds Ratio; CI, Confidence Interval; Ref, Reference category. The dependent variable was defined as binary awareness of nicotine pouches (Yes/No). The multivariable model is adjusted for all covariates listed in the table.

**Table 5:** Multivariable predictors of current nicotine pouch use.

Variable	*B*	aOR	95% CI for aOR	*p*-value
Age	−0.609	0.544	0.418–0.708	<0.001
Gender (male)	3.096	22.118	12.575–38.904	<0.001
Nationality (Saudi)	−0.027	0.973	0.421–2.251	0.950
Higher educational level	0.111	1.118	0.930–1.344	0.235
Higher monthly income	0.314	1.369	1.111–1.687	0.003
Employment (yes)	0.449	1.567	1.118–2.196	0.009
Marital status (married)	−0.564	0.569	0.370–0.874	0.010
Chronic disease	0.149	1.160	0.726–1.853	0.534

aOR, Adjusted Odds Ratio; CI, Confidence Interval; Ref, Reference category. The dependent variable was defined as current use of nicotine pouches (Yes/No). The multivariable model is adjusted for all covariates listed in the table.

In terms of awareness, age and gender emerged as highly significant predictors (Table 4). Each unit increase in age category was associated with a decrease in the odds of awareness (aOR = 0.452, 95% CI: 0.247–0.830, *p* = 0.010). Gender had a strong influence, with males being over seven times more likely to have higher awareness compared to females (aOR = 7.610, 95% CI: 2.462–23.529, *p* < 0.001). The geographic region also heavily influenced awareness. Compared to the Eastern region (reference), participants from the Western (aOR = 4.226, *p* = 0.015), Central (aOR = 3.653, *p* = 0.039), and Southern (aOR = 8.971, *p* = 0.049) regions had significantly higher odds of awareness. Interestingly, a reported smoking duration of up to 15 years was associated with lower odds of awareness (aOR = 0.589, *p* = 0.014).

Regarding predictors of NP use, younger age was significantly associated with higher odds of using NPs (aOR = 0.544, 95% CI: 0.418–0.708, *p* < 0.001) (Table 5). Male gender was significantly associated with higher odds of current nicotine pouch use compared to females (aOR = 22.118, 95% CI: 12.575–38.904, *p* < 0.001). Furthermore, socioeconomic variables emerged as significant predictors; both higher monthly income (aOR = 1.369, 95% CI: 1.111–1.687, *p* = 0.003) and active employment (aOR = 1.567, 95% CI: 1.118–2.196, *p* = 0.009) independently predicted higher odds of NP use. Conversely, being married was associated with lower odds of using NPs (aOR = 0.569, 95% CI: 0.370–0.874, *p* = 0.010) relative to unmarried participants.

## 4 Discussion

This study is among the first to comprehensively investigate awareness, usage patterns, and attitudes toward NPs among Saudi adults. Among the 1,004 participants, nearly 70% had heard of NP, yet comprehensive knowledge regarding their composition and regulatory aspects remains limited. NP use was reported by 25.5% of participants, with some showing dual-use behavior. Attitudes were mixed; although many participants recognized the appeal of NP, such as perceived safety and discretion, a majority expressed concern about their addictiveness and long-term risks. Younger males exhibited the highest rates of awareness and use. The same demographic also demonstrated Highly favorable attitudes, along with ex-smokers and current NP users.

Our findings indicate that approximately 70% of participants were aware of NPs, suggesting a relatively high level of public exposure to these products in Saudi Arabia. This finding aligns with a study conducted by Elsokkary et al. in the Riyadh region, where 75% of participants were aware of NPs (19). In contrast, global studies have reported significantly lower levels of awareness, with rates ranging between 24 and 40.6% (14, 15, 20). This may be attributed to differences in product availability and regulatory environments across countries.

In the Kingdom, NPs are sold via retail shops, local markets, and official brand websites, where they are commonly promoted for smoking cessation (13). Additionally, given the strong sociocultural context in the country, individuals may learn about NPs from family or friends, particularly if those individuals use NPs themselves as an attempt to quit smoking. This is supported by our finding that the most common sources from which participants heard about NPs were social media (44.5%) and family or friends (44.1%), in line with a previous study (15). In other countries, NPs might be less visible due to several reasons, including advertising restrictions, limited retail availability, or low user prevalence, culminating in individuals being less likely to encounter or recognize these products in daily life (21).

Although most participants were aware of NPs, comprehensive knowledge about their composition and regulatory aspects was lacking. While many respondents were aware that NPs are available in various flavors and strengths, awareness about their tobacco-free nature and age restriction regulations was limited. This suggests that public awareness is predominantly driven by marketing strategies that often promote features to enhance the appeal of the product, such as flavor variety, convenience, and discretion. Similar marketing trends were reported in the U.S., where promotional efforts often emphasize flavors, convenience, and discretion to appeal to consumers, particularly younger demographics (4, 5). This partial knowledge is particularly concerning given the increasing availability of NPs and their rising appeal among younger individuals. Despite NPs being legal and regulated, with required health warnings and an age restriction of 18 years (22), the observed gap between their appeal and the limited public understanding of these regulations may hinder public health efforts and potentially lead to product misuse by underage individuals.

In this study, 25.5% of participants reported using NPs, a prevalence notably higher than numbers reported in previous studies. For example, studies from the USA reported prevalence rates ranging from 3.8 to 10.3% (15, 23–25), while studies from Poland and the United Kingdom reported similarly low rates of 9.2 and 4.4%, respectively (14, 26). In contrast, our findings are nearly identical to a study conducted in Riyadh (19), which reported a prevalence of 20.5%. A possible contributor to this high rate may be “Badael,” a government-supported initiative aimed at improving quality of life and sustainability, which promotes locally produced NPs as safer and cleaner alternatives for smoking cessation (13). This aligns with our finding that smoking cessation was the primary reason for continued NP use, consistent with a previous study (15). Nevertheless, a substantial subset of users reported other motivations such as recreational nicotine addiction and stress relief. These motives raise concerns about whether NPs are being genuinely used for smoking cessation or simply as an alternative source of sustained nicotine dependence.

Approximately 40% of users reported dual use of NPs with other nicotine products, and nearly one-third acknowledged experiencing cravings, suggesting a pattern of use associated with increased nicotine dependence. This finding mirrors previous research in which NPs were combined with cigarettes or other nicotine-containing products (21). This dual-use pattern may indicate that NPs are utilized as a substitute in situations where conventional tobacco use is prohibited (27), benefiting from their discreet and convenient nature, rather than representing a full transition away from tobacco products.

Sociodemographic factors influencing awareness and use were observed, with age and gender being strong predictors. Young individuals aged between 18 and 35 years consistently demonstrated higher levels of awareness and use compared to older age groups. The present findings align with prior global (14, 15, 23, 24, 28) and regional (19) studies. Gender differences were noted, with males demonstrating higher awareness and use of NPs than females, aligning with prior studies (19, 24, 25). This is expected given the context of Saudi Arabia, where females are typically less likely to be interested or engage with nicotine-containing products, reflecting sociocultural factors associated with gender norms of the country (29).

Attitudes toward NPs among participants were ambivalent, encompassing both favorable and unfavorable perspectives. The perceived attractiveness of NPs was mainly driven by their variety of flavors, lack of smell, convenience, and discreet use. These features can enhance social acceptability and position NPs as “lifestyle-friendly” products. The availability of highly flavored NPs has been shown to encourage experimentation, particularly among novice users (30), which could potentially heighten the risk of product misuse. In the context of Saudi Arabia, the risk may be amplified by the introduction of region-specific flavors, such as Bonna, with a coffee aroma from Jazan, and Unwood, with a grape scent from Taif (13). This localized marketing approach, combined with the concentration of retail distribution in major metropolitan and commercial hubs within the Central and Western regions, likely explains our finding that participants from these specific geographic areas demonstrated significantly higher odds of awareness. Although females were not as likely as males to be aware of or use NPs in our study, these appealing features, particularly flavors and odorlessness, could play a role in attracting female users, as seen in a previous study (31).

Perceived harm reduction of NPs was expressed by participants. Many perceive NPs to be safer than traditional tobacco products or e-cigarettes and can help people quit smoking. These perceptions are largely influenced by marketing claims such as being “less harmful” or “tobacco-free” alternatives (5). Prior studies have demonstrated that holding such favorable beliefs is associated with increased susceptibility to NP use (24, 32). In our study, young adults aged between 26 and 35 and ex-smokers shared similar favorable attitudes to those of current NP users, suggesting that these groups may be at increased risk of future uptake.

Nonetheless, health concerns were prominent. Many participants expressed concerns about the addictive potential of NPs, associated oral health risks, and their unknown long-term health effects. These risks are exacerbated due to the availability of high nicotine strengths, which may lead to nicotine addiction (33). Although long-term health outcomes remain unknown due to the novelty of NPs, the high nicotine content in these products is expected to increase the risk of cardiovascular, respiratory, and gastrointestinal disorders (34). In addition, their mode of use can cause mucosal compression, potentially inducing local inflammation and ulceration of the oral mucosa (33, 35). Given these concerns, many participants supported stricter regulations on NPs, reflecting a public health demand for stronger safeguards to prevent youth uptake and mitigate potential long-term harms.

This study has several limitations that should be considered when interpreting the findings. First, its cross-sectional nature precludes inferences of causality, and the use of self-report data could result in recall and social desirability biases. Second, adolescents were not included in the study, and future research is warranted to explore the susceptibility to NP use in this age group. Third, the use of the convenience sampling method using social media may have led to a sample of younger, digitally active individuals, and could limit the generalizability of the results. Fourth, the study did not consider clinical or objective markers of nicotine exposure, nor did it assess symptoms or signs associated with NP use. Thus, future research incorporating these measures could enhance validity and clinical relevance. Fifth, sparse data from the low number of female users inflated the odds ratio and confidence interval for male gender, warranting cautious interpretation of its exact magnitude. Sixth, some data points may have been incomplete due to the nature of online surveys and the self-administration format. Furthermore, the use of a broad ‘Over 45 years’ age bracket limits our ability to distinguish potential behavioral or attitudinal differences between middle-aged and older adults. Finally, most participants were from the Eastern region, potentially limiting the representativeness of findings across other regions of Saudi Arabia. Despite these limitations, the study provides important insights on NP awareness, use, and attitudes, and can serve as a foundation for future research employing longitudinal and more representative designs.

## 5 Conclusion

Awareness and use of NPs are rising among young individuals in Saudi Arabia, although notable knowledge gaps persist regarding their composition and regulation. While many users primarily use NPs for smoking cessation, dual use with other nicotine products and reported cravings indicate potential for sustained nicotine dependence. These findings necessitate targeted public health strategies to address misconceptions, alongside evidence-based regulatory measures to balance potential harm-reduction benefits with the prevention of misuse, especially among non-smokers and youth.

## Data Availability

The original contributions presented in the study are included in the article/Supplementary material, further inquiries can be directed to the corresponding author.

## Supplementary material

SUPPLEMENTARY FILE 1 Questionnaire.

SUPPLEMENTARY FILE 2 STROBE checklist.
