# Correction: Assessment of awareness, usage, and attitudes toward nicotine pouches in Saudi Arabia: a cross-sectional study

**DOI:** 10.3389/fpubh.2026.1987452

**Published:** 2026-09-23

**Authors:** Hussain Khalifa Aljumah, Hassan Ali Alradhi, Mujtaba Abbas Alzuwayr, Manal Yousef Alturaifi, Maryam Ali Alamer, Mohammed Naji Albannay, Abdullah Almaqhawi

**Affiliations:** 1 College of Medicine, King Faisal University, Al-Ahsa, Saudi Arabia; 2 Faculty of Medicine, Jazan University, Jazan, Saudi Arabia; 3 College of Applied Medical Sciences, King Saud bin Abdulaziz University for Health Sciences, Al-Ahsa, Saudi Arabia; 4 Department of Family and Community Medicine, College of Medicine, King Faisal University, Al-Ahsa, Saudi Arabia

**Keywords:** attitudes, awareness, nicotine pouches, Saudi Arabia, smokeless tobacco, use

Reference (36) was not cited in the article. The citation has now been inserted in the section 1 **Introduction**, Paragraph 1 and should read: “Globally, SLT remains a major public health threat, accounting for an estimated 350,000 deaths annually worldwide (36).”

In the published article, there was an error in the affiliations for King Faisal University. Instead of “Al Hofuf”, it should be “Al-Ahsa”.

A correction has been made to the author affiliations:

“^1^College of Medicine, King Faisal University, Al-Ahsa, Saudi Arabia, ^2^Faculty of Medicine, Jazan University, Jazan, Saudi Arabia, ^3^College of Applied Medical Sciences, King Saud bin Abdulaziz University for Health Sciences, Al-Ahsa, Saudi Arabia, ^4^Department of Family and Community Medicine, College of Medicine, King Faisal University, Al-Ahsa, Saudi Arabia”.

In the published article, references 14 and 26 were incorrectly cited alongside USA prevalence rates.

A correction has been made to the section **4 Discussion**, Paragraph 5:

“For example, studies from the USA reported prevalence rates ranging from 3.8 to 10.3% (15, 23–25), while studies from Poland and the United Kingdom reported similarly low rates of 9.2 and 4.4%, respectively (14, 26).”

In the published article, the in-text callouts for Supplementary Files 1 and 2 were reversed.

A correction has been made to the section **2 Methodology**, subsection **2.3 Data collection tool**, Paragraph 1:

“The questionnaire was adapted from previously published studies on novel nicotine products (**Supplementary File 1**) (14–17).”

A correction has also been made to the section **2 Methodology**, subsection **2.6 Ethical considerations**, Paragraph 1:

“The study adhered to Strengthening the Reporting of Observational Studies in Epidemiology (STROBE) guidelines for cross-sectional studies (**Supplementary File 2**) (18).”

The original version of this article has been updated.

