# Supplementary material for: Assessment of awareness, usage, and attitudes toward nicotine pouches in Saudi Arabia: a cross-sectional study
Source: Front Public Health. 2026 Aug 27;14:1896313. doi: 10.3389/fpubh.2026.1896313 (PMC13558216; doi:10.3389/fpubh.2026.1896313)
Supplement: Supplementary file 1 [file Supplementary_file_1.docx]

**Assessment of Awareness, Usage, and Attitudes Toward Nicotine Pouches in Saudi Arabia: A Cross-Sectional Study**

Greetings,

We are a group of researchers from several universities in the Kingdom of Saudi Arabia. We invite you to fill out this questionnaire, which aims to assess the level of awareness regarding the use of nicotine pouches in Saudi Arabia.

Participation in this study is voluntary, and responses will remain anonymous.
All data will be used for research purposes only.
Ethical approval has been obtained from King Faisal University under the reference: KFU-REC-2025-MAR-ETHICS3222.

Would you like to participate in this study?
- Yes
- No

## Section One: General Information

1. Age
- 18–25 years
- 26–35 years
- 36–45 years
- Over 45 years

2. Gender
- Male
- Female

3. Nationality
- Saudi
- Non-Saudi

4. Educational level
- Up to Secondary Education
- Bachelor’s degree
- Master’s/PhD

5. Residential region
- Eastern Region
- Central Region
- Western Region
- Northern Region
- Southern Region

6. Employment status
- Employed
- Unemployed
- Student
- Retired

7. Marital status
- Married
- Divorced
- Single

8. Monthly income
- Less than 5,000 SAR
- 5,000–10,000 SAR
- 10,000–15,000 SAR
- More than 15,000 SAR

9. Do you suffer from any chronic illnesses? (Check all that apply)
- I do not have any chronic illnesses
- Yes, diabetes
- Yes, chronic hypertension
- Yes, chronic obstructive pulmonary disease (COPD)
- Other: _____

10. Are you currently a smoker?
- Yes, I smoke cigarettes
- Yes, I use electronic cigarettes (e.g., vape)
- Yes, I smoke shisha
- No, I used to smoke
- I have never smoked

11. If yes, for how long have you been smoking?
- Less than 5 years
- 5–10 years
- 11–15 years
- More than 15 years

## Section Two: Awareness About Nicotine Pouches

1. Have you heard of smokeless tobacco products (e.g., snus or moist snuff)?
- Yes
- No

2. Have you heard of nicotine pouches (e.g., Fox, DZRT, Velo)?
- Yes
- No

3. If yes, where did you hear about nicotine pouches?
- Family / Friends
- Social media
- Advertisements
- Healthcare provider
- Other sources

4. Did you know that nicotine pouches come in different flavors and strengths?
- Yes
- No

5. Did you know that nicotine pouches are tobacco-free?
- Yes
- No

6. Are you aware of age restrictions for purchasing and consuming nicotine pouches?
- Yes
- No

## Section Three: Usage

1. Do you use nicotine pouches?
- Yes
- No
*(Instruction: If you answered "No" to the first question, please select “I am not a user of nicotine pouches” for the rest of this section and proceed directly to Section Four.)*
2. If yes, how many pouches do you use daily on average?
- Less than 5 pouches
- 5–10 pouches
- More than 10 pouches

3. How likely are you to continue using nicotine pouches over the next year?
- Very likely
- Likely
- Neutral
- Unlikely
- Very unlikely

4. What is your main reason for continuing to use nicotine pouches?
- To quit smoking
- Recreational use / nicotine addiction
- To relieve stress or depression
- Taste
- Other

5. Do you combine nicotine pouches with other nicotine products (cigarettes, vape, etc.)?
- Yes
- Sometimes
- No

6. Does using nicotine pouches make you crave more pouches?
- Yes
- Sometimes
- No

## Section Four: Attitudes Toward Nicotine Pouches

*Please rate your agreement with the following statements (Based on a 5-point Likert scale where 5 = Strongly Agree, and 1 = Strongly Disagree). ^(R)^ Items were reversed-scored in the final analysis.*

1. I believe nicotine pouches are addictive. ^(R)^
2. I believe nicotine pouches are safer than cigarettes and shisha.
3. I believe nicotine pouches are safer than electronic cigarettes.
4. I believe nicotine pouches can help people quit smoking.
5. I believe nicotine pouches are more socially accepted than smoking.
6. I believe the long-term health effects of nicotine pouches are not well understood. ^(R)^
7. I believe nicotine pouches are easier to use and more discreet than regular tobacco products.
8. I believe the variety of flavors makes nicotine pouches more attractive to consumers.
9. I believe nicotine pouches can be misused by non-smokers. ^(R)^
10. I believe nicotine pouches can cause gum disease or other oral health problems. ^(R)^
11. I believe the popularity of nicotine pouches is due to their lower cost compared to other tobacco products.
12. I believe the lack of smell makes nicotine pouches more attractive to some people.
13. I believe stricter regulations should be enforced on nicotine pouches. ^(R)^

**تقييم الوعي والاستخدام والمواقف تجاه أظرف النيكوتين في المملكة العربية السعودية: دراسة مقطعية**

السلام عليكم ورحمة الله وبركاته، تحية طيبة للجميع. نحن مجموعة من الباحثين من عدة جامعات في المملكة العربية السعودية. ندعوكم إلى تعبئة هذا الاستبيان الذي يهدف إلى تقييم مستوى الوعي حول استخدام أظرف النيكوتين في المملكة العربية السعودية.

المشاركة في هذه الدراسة اختيارية والردود ستكون مجهولة الهوية تماماً. جميع البيانات ستُستخدم لأغراض بحثية فقط. تم الحصول على الموافقة الأخلاقية من جامعة الملك فيصل تحت المرجع: KFU-REC-2025-MAR-ETHICS3222

هل ترغب بالمشاركة في الدراسة؟

- نعم
- لا

**القسم الأول: المعلومات العامة**

1. العمر

- 18 - 25 سنة
- 26 - 35 سنة
- 36 - 45 سنة
- أكبر من 45 سنة

2. الجنس

- ذكر
- أنثى

3. الجنسية

- سعودي
- غير سعودي

4. المستوى التعليمي

- التعليم الثانوي وما دون
- تعليم متوسط / دبلوم
- بكالوريوس
- ماجستير / دكتوراه

5. المنطقة السكنية

- المنطقة الشرقية
- المنطقة الوسطى
- المنطقة الغربية
- المنطقة الشمالية
- المنطقة الجنوبية

6. الحالة الوظيفية

- موظف
- غير موظف
- طالب
- متقاعد

7. الحالة الاجتماعية

- متزوج
- مطلق / أرمل
- أعزب

8. الدخل الشهري

- أقل من 5000 ريال سعودي
- 5000 - 10000 ريال سعودي
- 10000 - 15000 ريال سعودي
- أكثر من 15000 ريال سعودي

9. هل تعاني من أي أمراض مزمنة؟ (اختر كل ما ينطبق)

- لا أعاني من أي أمراض مزمنة
- نعم، مرض ارتفاع ضغط الدم المزمن
- نعم، مرض السكري
- نعم، الربو
- نعم، مرض الانسداد الرئوي المزمن
- أخرى: _____

10. هل أنت مدخن حاليًا؟

- نعم، أدخن السجائر
- نعم، أدخن السجائر الإلكترونية (مثل الفيب)
- نعم، أدخن الشيشة
- لا، كنت مدخنًا سابقًا
- لم أدخن أبدًا

11. إذا كانت الإجابة نعم، منذ متى وأنت تدخن؟

- أقل من 5 سنوات
- 5 - 10 سنوات
- 11 - 15 سنة
- أكثر من 15 سنة

**القسم الثاني: الوعي بأظرف النيكوتين**

1. هل سمعت من قبل عن منتجات التبغ غير المدخن (مثل النشوق أو التبغ الرطب)؟

- نعم
- لا

2. هل سمعت من قبل عن أظرف النيكوتين مثل (DZRT, Velo, Fox)؟

- نعم
- لا

3. إذا كانت الإجابة نعم، من أين سمعت عن أظرف النيكوتين؟

- العائلة / الأصدقاء
- وسائل التواصل الاجتماعي
- إعلانات
- مقدم رعاية صحية
- مصادر أخرى

4. هل تعلم أن أظرف النيكوتين تتوفر بنكهات وقوى تركيز مختلفة؟

- نعم
- لا

5. هل تعلم أن أظرف النيكوتين خالية من التبغ؟

- نعم
- لا

6. هل أنت على علم بالقيود العمرية المتعلقة بشراء واستهلاك أظرف النيكوتين؟

- نعم
- لا

**القسم الثالث: استخدام أظرف النيكوتين**

1. هل تستخدم أظرف النيكوتين؟

- نعم
- لا

*إرشاد: إذا كانت إجابتك "لا" على السؤال الأول، يرجى اختيار" لست من مستخدمي أظرف النيكوتين" للأسئلة في باقي هذا القسم والانتقال مباشرة إلى القسم الرابع.*

2. كم عدد الأظرف التي تستهلكها يوميًا بشكل تقريبي؟

- أقل من 5 أظرف
- 5 - 10 أظرف
- أكثر من 10 أظرف

3. ما مدى احتمالية استمرارك في استخدام أظرف النيكوتين خلال العام القادم؟

- عالي الاحتمال
- محتمل
- محايد
- غير محتمل
- قليل الاحتمال

4. ما هو السبب الأساسي للاستمرار في استخدام أظرف النيكوتين؟

- للإقلاع عن التدخين
- للاستخدام الترفيهي / إدمان النيكوتين
- لتخفيف التوتر والاكتئاب
- المذاق
- لا يوجد سبب محدد

5. هل تجمع بين استخدام أظرف النيكوتين ومنتجات النيكوتين الأخرى (السجائر، فيب، الخ)؟

- نعم
- أحيانًا
- لا

6. هل يجعلك استخدام أظرف النيكوتين تشعر برغبة في استهلاك أظرف أكثر؟

- نعم
- أحيانًا
- لا

**القسم الرابع: المواقف تجاه أظرف النيكوتين**

*يرجى تقييم مدى موافقتك على العبارات التالية (بناءً على مقياس ليكرت من 5 نقاط حيث 5 = أوافق بشدة، و 1 = لا أوافق بشدة). ^(R)^ تم عكس درجات هذه الفقرات في التحليل النهائي.*

1. أعتقد أن أظرف النيكوتين تسبب الإدمان^(R)^ .
2. أعتقد أن أظرف النيكوتين أكثر أمانًا من السجائر والشيشة.
3. أعتقد أن أظرف النيكوتين أكثر أمانًا من السجائر الإلكترونية.
4. أعتقد أن أظرف النيكوتين يمكن أن تساعد الأشخاص على الإقلاع عن التدخين.
5. أعتقد أن أظرف النيكوتين مقبولة اجتماعيًا أكثر من التدخين.
6. أعتقد أن التأثيرات الصحية طويلة المدى لأظرف النيكوتين غير مفهومة جيدًا^(R)^ .
7. أعتقد أن أظرف النيكوتين أسهل في الاستخدام وأكثر سرية من منتجات التبغ العادية.
8. أعتقد أن تنوع النكهات في أظرف النيكوتين يجعلها أكثر جاذبية للمستهلكين.
9. أعتقد أن أظرف النيكوتين يمكن أن يُساء استخدامها من قبل غير المدخنين^(R)^ .
10. أعتقد أن أظرف النيكوتين يمكن أن تسبب أمراض اللثة أو مشكلات صحية أخرى في الفم ^(R)^ .
11. أعتقد أن شعبية أظرف النيكوتين تعود إلى كونها أرخص من منتجات التبغ الأخرى.
12. أعتقد أن عدم وجود رائحة لأظرف النيكوتين يجعلها أكثر جاذبية للبعض.
13. أعتقد أنه يجب فرض قوانين أكثر صرامة على أظرف النيكوتين^(R)^ .
